# Chemical characterization, assessment of acute oral toxicity, and antinociceptive potential of the methanolic extract of *Montrichardia linifera* (Arruda) Schott leaves from Brazil

**DOI:** 10.3389/fphar.2024.1475157

**Published:** 2024-11-20

**Authors:** Wellington Junior Taisho Nagahama Costa, Leticia Prazeres de Farias Coelho, Alan Luz Tembra, Rayan Fidel Martins Monteiro, Jose Ramon Gama Almeida, Klinsmann Thiago Lima, Anderson de Santana Botelho, Raimundo Junior da Rocha Batista, Jofre Jacob da Silva Freitas, Wandson Braamcamp de Souza Pinheiro, Fabiola Raquel Tenorio Oliveira, Karen Renata Herculano Matos Oliveira, Anderson Bentes de Lima, Cristine Bastos do Amarante, Gilmara de Nazareth Tavares Bastos

**Affiliations:** ^1^ Laboratory of Neuroinflammation, Institute of Biological Sciences, Federal University of Pará, Belém, Brazil; ^2^ Laboratory of Morphophysiology Applied to Health, Department of Morphology and Physiological Sciences, State University of Pará, Belém, Brazil; ^3^ Laboratory of Chemical Analysis, Coordination of Earth Sciences and Ecology, Emílio Goeldi Museum, Belém, Brazil; ^4^ Laboratory of Central Extraction, Institute of Exact and Natural Sciences, Federal University of Pará, Belém, Brazil; ^5^ Laboratory of Experimental Neuropharmacology, Institute of Biological Sciences, Federal University of Pará, Belém, Brazil; ^6^ Laboratory of Developmental Biology, Department of Morphology, Federal University of São Paulo, São Paulo, Brazil

**Keywords:** acute oral toxicity, antinociceptive, flavonoid, medicinal plant, *Montrichardia linifera*

## Abstract

**Background:**

*Montrichardia linifera* (Arruda) Schott is popularly known as “aninga,” “aningaçu,” “aningaíba,” and “aninga-do-igapó.” Compresses and plasters made from the leaves of this medicinal plant are used to treat abscesses, tumors, and pain caused by stingray stings.

**Aim of the study:**

This study aimed to chemically characterize the methanolic extract of *M. linifera* leaves (MEMLL), as well as to verify their acute oral toxicity and antinociceptive potential.

**Materials and methods:**

The leaves were collected during the rainy season, and the methanolic extract was obtained after gradient extraction using different solvents. MEMLL was analyzed using high-performance liquid chromatography (HPLC) and nuclear magnetic resonance (NMR). Acute oral toxicity testing followed the Organization for Economic Co-operation and Development (OECD) guideline 423. Subsequently, acetic acid, hot plate, and formalin tests were used to evaluate the analgesic effects.

**Results:**

In the chemical characterization of MEMLL by HPLC, three flavonoids were identified: rutin, quercetin, and epicatechin. In addition, when NMR spectroscopy was performed, rutin and quercetin were again identified, as well as the chemical compounds luteolin and chrysoeriol. In the acute oral toxicity test, MEMLL showed no physiological or behavioral changes. In the nociceptive study, MEMLL showed an effect at doses of 50 and 100 mg/kg in the 0.6% acetic acid test, i.e., 51.46% and 75.08%, respectively. In the hot plate test, the MEMLL group at a dose of 50 mg/kg was effective at times of 30 and 60 min, i.e., 164.43% and 122.95%, respectively. Similarly, the MEMLL group at a dose of 100 mg/kg was also effective in increasing latency at times of 30 and 60 min, i.e., 162.62% and 136.68%, respectively. In the formalin test, MEMLL showed an antinociceptive effect on neurogenic pain at doses of 50 and 100 mg/kg when compared to the control group, 35.25% and 52.30%, respectively. In the inflammatory phase, inhibition was observed in the MEMLL at doses of 50 and 100 mg/kg, i.e., 66.39% and 72.15%, respectively.

**Conclusion:**

MEMLL has analgesic properties and is non-toxic, validating the Brazilian ethnopharmacological use of this plant for pain treatment. The leaves of the species *M. linifera* showed central and peripheral antinociceptive effects.

## 1 Introduction

Pain affects not only wellbeing but also the health and productivity of individuals, a growing public health concern that has become a global problem (Del Gaudio et al., 2023). In many diseases, pain serves as a warning system amongst noxious stimuli, a defense mechanism when something in the body is not working properly (Vargas-Ruiz et al., 2023). It is the main reason people seek medical treatment (Cohen et al., 2021). Pain is currently defined as “an unpleasant sensory and emotional experience associated with, or resembling that associated with, actual or potential tissue damage,” according to the International Association for the Study of Pain (IASP) (Raja et al., 2020).

Pharmacological intervention is the most widely used treatment for pain relief among several treatments. It includes the use of non-steroidal anti-inflammatory drugs (NSAIDs) and opioids for peripheral and central analgesic action, respectively (Marinho et al., 2023). These are potentially effective in the treatment of pain, but the administration of these drugs often causes adverse effects (Nascimento et al., 2024). The use of NSAIDs can cause gastrointestinal bleeding and renal dysfunction, and opioids are associated with potentially fatal respiratory depression, dependence, and constipation (Jabbari et al., 2024). Thus, there is a current need to find new analgesic agents (Del Gaudio et al., 2023).

Natural products serve as a source of several therapeutic agents that constitute modern medical practice (Ema et al., 2023). They have gained prominence for their promising effects in treating diseases, resulting from numerous chemical compounds, the most successful source of potential medicines (Marinho et al., 2023; Pires et al., 2024). The World Health Organization (WHO) estimates that traditional medicine, plant extracts, or their active ingredients are used to meet primary health needs and improve the quality of life in 80% of the world’s population (Del Gaudio et al., 2023). So, medicinal plants have emerged as an alternative for treating diseases in folk medicine since ancient civilizations (Santana et al., 2023), playing an important role in scientific research to reduce side effects and regulate pain (Nascimento et al., 2024).

In this context, Brazil has a great capacity to develop studies with medicinal plants, being the country with the most incredible plant diversity, with around 20% of the world’s flora (Ferreira et al., 2022). *Montrichardia linifera* (Arruda) Schott is a species of aquatic macrophyte native and non-endemic to Brazil (Miranda et al., 2015), belonging to the genus *Montrichardia Crueg* of the Araceae family (Lopes et al., 2016). It is popularly known as “aninga,” “aningaçu,” “aningaíba,” and “aninga-do-igapó” (Costa et al., 2009).

Its sap is traditionally used in Brazilian ethnopharmacology as a healing, antirheumatic, antidiuretic, and expectorant medicine. However, its excessive use can be considered toxic due to its ability to cause burns, skin eruptions, spots, and, in the case of eye contact, blindness (Lima et al., 2021). The powder from the root of this plant is used as an antifungal medicine and anesthetic against stingray stings (Batista et al., 2019). The compresses and plasters made from the leaves are used to treat abscesses, tumors, and also against stingray stings (Amarante et al., 2011). In a previous phytochemical study, Faria et al. (2021) proved the methanolic extract of the leaves to be most promising for biological studies as the presence of groups of secondary metabolites such as coumarins, flavonic heterosides, tannins, polyphenols, and saponins was observed. Despite this, not many studies in the scientific literature demonstrate its biological potential.

Based on its traditional use, the current study aimed to investigate the analgesic effects of the methanolic extract of *M. linifera* leaves (MEMLL). For this purpose, chemical characterization, acute oral toxicity test, and nociceptive tests were performed on MEMLL.

## 2 Materials and methods

### 2.1 Collection and extraction of the plant material

The *M. linifera* (Arruda) Schott leaves were collected during the rainy season from January to April 2015 in the city of Belém, Pará, Brazil (coordinates: 1°27′53.0″S, 48°30′24.3″W). The specimen was registered in the National Management System of Genetic Heritage and Associated Traditional Knowledge (SisGen, Brazil) under the registration number A91B68B, and the exsiccata was deposited in the João Murça Pires Herbarium of the Emílio Goeldi Museum under the registration number MG 216695.

The collected leaves were washed with running water and deionized water, cut into small pieces, and dried in a refrigerated environment at 16°C with 35% humidity. Afterward, drying was completed in an oven at 45°C, and the samples were ground in an industrial blender to obtain leaf powder (1,000 g) and subjected to gradient extraction (5 L for each solvent) in an increasing order of polarity (hexane, ethyl acetate, and methanol). The extracts were filtered and concentrated in a rotary evaporator model Q344M2 (QUIMIS^®^; São Paulo, Brazil) using an ultra-thermostatic bath model Q214M2 (QUIMIS^®^; São Paulo, Brazil) and then dried in an oven at 45°C.

### 2.2 Chemical characterization of MEMLL

MEMLL was characterized by adapting the methods described by Williams et al. (2015), Singh and Dhepe (2018), Tomazi et al. (2021), Pinheiro et al. (2022), and Kuhn and Nuzillard (2023). The organic compounds were identified using the following analytical techniques: high-performance liquid chromatography (HPLC) and nuclear magnetic resonance (NMR) spectroscopy.

#### 2.2.1 Chromatographic profile by HPLC

A clean-up was carried out on the investigated MEMLL to remove hydrophobic pigments, such as chlorophylls. For this purpose, these interferents were removed by solid-phase extraction (SPE). Initially, C18 SPE cartridges (1 g/6 mL; Strata, Phenomenex) were cleaned and activated with water and acetonitrile. Subsequently, 180 mg of MEMLL solubilized in water/acetonitrile (30:70, v/v) was injected and extracted with two volumes in the same ratio. After obtaining the extracting solutions, they were dried in an oven with air circulation.

The chromatographic analysis by HPLC of the MEMLL was performed in a liquid chromatograph Prominence LC-20AT model (Shimadzu, Tokyo, Japan) with a diode array detector (DAD) and Gemini C18 column (250 mm × 4.6 mm, 5 μm) (Phenomenex; Torrance, CA, United States). LCSolution 1.20 software was used for data processing. Then, 20 µL/injection of standardized MEMLL was applied at 1,000 ppm and eluted in a water/acetonitrile gradient, with an organic modifier ranging linearly from 5% to 100%, in 60 min and a flow rate of 1 mL/min, according to the method described by Pinheiro et al. (2022). MEMLL was monitored from the compound’s absorbance under ultraviolet radiation, from 200 to 400 nm.

To obtain fingerprinting, the retention times (t_r_), ultraviolet spectra, and NMR data on the flavonoid standards epicatechin, rutin, quercetin, myricetin, and kaempferol were compared with those observed for the MEMLL.

#### 2.2.2 Spectroscopy profile by NMR

The NMR spectra of ^1^H, ^13^C, and the correlation maps homonuclear correlation spectroscopy (HOMO-COSY), heteronuclear single quantum coherence (HSQC), and heteronuclear multiple-bond coherence (HMBC) were obtained on a Bruker spectrometer, Ascend™ (Rheinstetten, Germany) model, operating at 400 and 100 MHz. Preparation involved dissolving 50 mg of MEMLL in 600 µL of deuterated dimethyl sulfoxide (DMSO-d6), with subsequent data control and processing done using TopSpin software (version 3.6.0). The free induction decays (FIDs) were transformed using Fourier transform, applying a line broadening (LB) of 0.3 Hz to enhance the resolution. The manual adjustments were made for baseline correction and calibration using the residual solvent peak as an internal reference, DMSO at 2.49 (^1^H) and 39.5 ppm (^13^C), according to the adaptation of the methods proposed by Williams et al. (2015), Singh and Dhepe (2018), Tomazi et al. (2021), and Kuhn and Nuzillard (2023).

The abundance of functional groups present in the classes of metabolites of interest was analyzed in the processing of FIDs, grouped, and normalized into specific regions (δ = 0.5–1.5/1.5–3.0/3.0–04.5/4.5–6.0/6.0–9.0/9.0–10.0) (Pinheiro et al., 2022). To indicate the selected peaks, chemical shift data (δ) and coupling constant (J) of one-dimensional spectra (1D) ^1^H e ^13^C were used, in addition to two-dimensional correlation maps 2D ^1^H ^1^H (HOMO-COSY), ^1^H^13^C (HSQC), and ^1^H^13^C (HMBC). The experimental data were compared with the respective literature signals for flavonoid structures.

### 2.3 Experimental animals and ethical statement

All animals in this research were treated according to the ethical guidelines for the care and use of laboratory animals. Eighty-eight adult male mice (*Mus musculus*; 30–40 g, 8–10 weeks old, Swiss) were obtained from the Laboratory Animal Breeding and Production Section of the Evandro Chagas Institute and retained in the “Luiz Carlos de Lima Silveira” animal facility of the State University of Pará. Before testing, the animals were acclimatized for 1 week, maintained under a 12-h light/dark cycle, controlled temperature of 22°C ± 2°C, and with access to food Nuvilab CR1 (NUVITAL Nutrientes Ltda., Curitiba, Brazil) and water available *ad libitum*. This research was approved by the State University of Pará Animal Use Ethics Committee under protocol 09/2017.

### 2.4 Acute oral toxicity test

The acute oral toxicity test was performed according to the Organization for Economic Co-operation and Development (OECD) guideline 423 (OECD, 2002). The mice were divided into three groups of six animals: the control group received a vehicle (1% Tween 80 diluted in saline solution, v/v, p.o.; n = 6) and two treatment groups (MEMLL at doses of 300 and 2.000 mg/kg, p.o.; n = 6 per group). A single oral dose was administered individually to each animal. Subsequently, they were observed for 4 h and then every 24 h for 14 days to evaluate possible behavioral and physiological changes such as aggression, alertness, apathy, ataxia, convulsions, diarrhea, lack of appetite, locomotion, nasal secretion, piloerection, response to touch, spontaneous motor activity, stereotypy, sweating, and urination.

During this period, body weight gain and food and water consumption were recorded. On day 14, the mice were subjected to induced euthanasia (300 mg/kg ketamine and 30 mg/kg xylazine) for the macroscopic evaluation of internal organs (the liver, kidney, and spleen) and masses obtained using an analytical balance.

### 2.5 Evaluation of antinociceptive activity

#### 2.5.1 Acetic acid-induced writhing test

The experiment in mice was performed according to the method reported by Koster et al. (1959). Abdominal writhing was induced in animals by intraperitoneal administration of 0.6% acetic acid in a volume of 10 mL/kg. The mice were divided into six groups of five animals: the control group received the vehicle (1% Tween 80 diluted in saline solution, v/v, p.o.; n = 5), four treatment groups (MEMLL at doses of 10, 25, 50, and 100 mg/kg, p.o.; n = 5), and a positive control group (5 mg/kg indomethacin, i.p.; n = 5). After 60 min of treatment, the animals received an intraperitoneal administration of 0.6% acetic acid, and then, the abdominal contortions and hind paw extensions were recorded for 30 min.

The doses that showed the best results in this experiment were chosen for the other nociception tests.

#### 2.5.2 Hot plate test

The central analgesic activity was evaluated using a model established by Macdonald et al. (1946). The animals were placed on a hot plate at a fixed temperature (55°C ± 0.5°C), and the latency time to respond to the temperature change, such as licking one of the hind paws or jumping, was recorded. The mice were divided into four groups of five animals: the control group received the vehicle (1% Tween 80 diluted in saline solution, v/v, p.o.; n = 5), two treatment groups (MEMLL at doses of 50 and 100 mg/kg, p.o.; n = 5), and a positive control group (10 mg/kg morphine, s.c.; n = 5). Subsequently, the animals were individually placed on the hot plate, and the response was evaluated at times 0, 30, 60, 90, and 120 min. The maximum latency time of 40 s was respected to avoid tissue damage.

#### 2.5.3 Formalin-induced licking response

To confirm the antinociceptive effect of the MEMLL, the formalin nociception test was performed according to the methodology described by Hunskaar et al. (1985). The mice were divided into four groups of five animals: the control group received the vehicle (1% Tween 80 diluted in saline solution, v/v, p.o.; n = 5), two treatment groups (MEMLL at doses of 50 and 100 mg/kg, p.o.; n = 5), and a positive control group (4 mg/kg morphine, s.c.; n = 5). After 60 min of treatment, the test involved an intraplantar injection of 20 µL of 1% formalin solution into the right hind paw of each animal. The time during which the animal licked its paw was recorded in the first phase from 0 to 5 min (neurogenic pain) and in the second phase from 15 to 30 min (inflammatory pain).

### 2.6 Statistical analysis

GraphPad Prism^®^ version 10.2.3 (GraphPad Software Inc., San Diego, CA, United States) was used to perform statistical tests. First, the data normality test (Shapiro–Wilk) was used to determine the type of statistical tests. Then, one-way analysis of variance (ANOVA) was performed on the results of the acute oral toxicity test, the writhing test, and the formalin-induced licking response test, and two-way ANOVA was performed on the hot plate test. All tests were followed by Dunnett’s multiple comparison *post hoc* test. The data are expressed as the mean ± standard error of the mean (SEM). Statistical differences were considered significant when *p* < 0.05.

## 3 Results

### 3.1 Chemical characterization of MEMLL

#### 3.1.1 Chromatographic profile by HPLC

**FIGURE 1:**
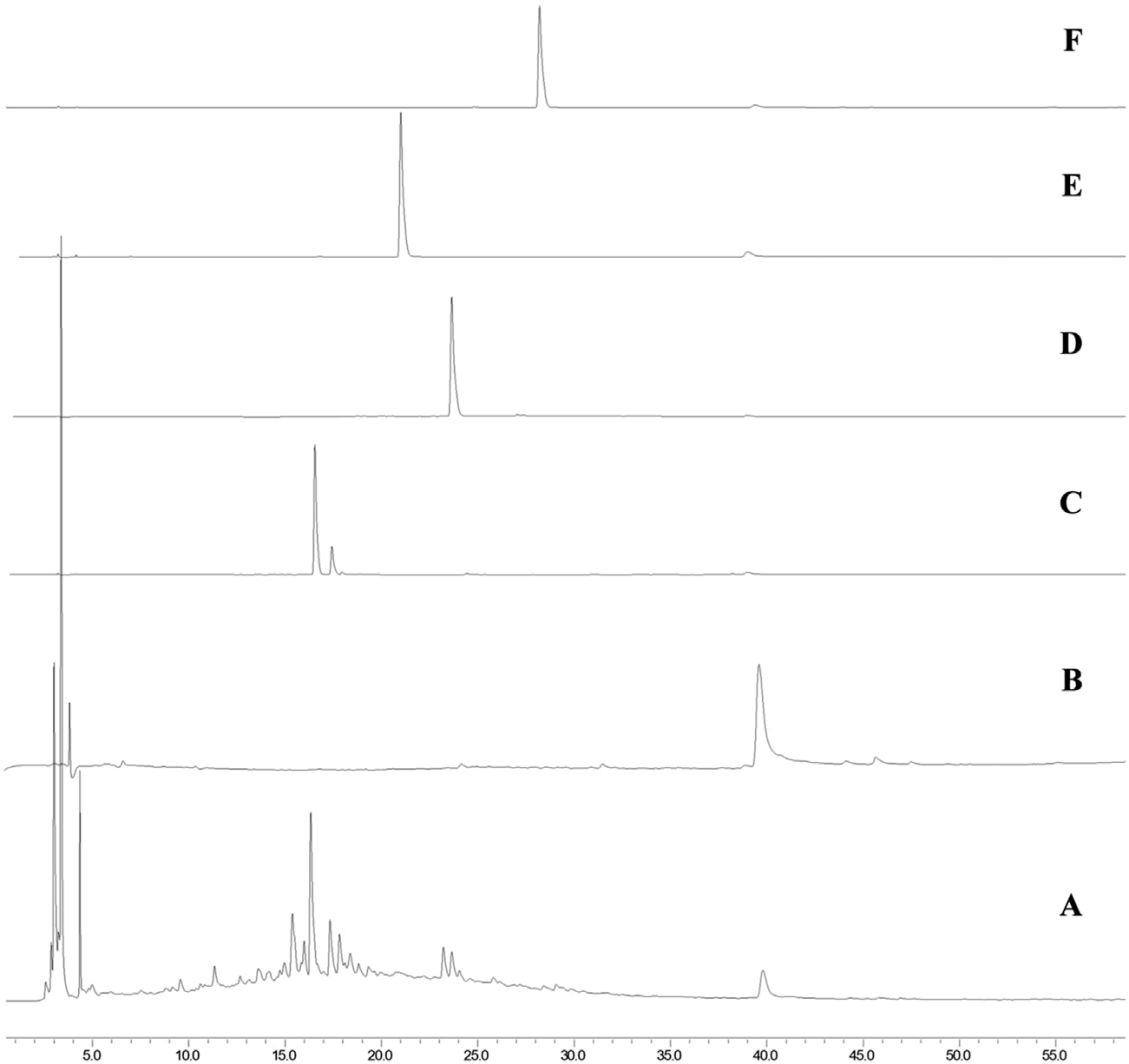
HPLC analysis. **(A)** MEMLL; **(B)** epicatechin; **(C)** rutin; **(D)** quercetin; **(E)** myricetin; **(F)** and kaempferol.

To obtain the initial information about the compounds present in MEMLL, the chromatographic profile was obtained by HPLC-DAD, as well as from the comparison of t_r_. The extract was fingerprinted against the flavonoid standards (Figure 1). In the chromatogram analysis of the extract at wavelength 270 nm, chromatographic bands of chromophoric substances were observed between 3.5 and 23 min, attributed to more polar and hydrophilic substances, and an isolated chromatographic band was also observed at 39.7 min. In the individual analysis of the ultraviolet spectra of each peak observed in the chromatogram, all bands showed the same spectral pattern, with absorption maxima at 220 and 270 nm or 220, 270, and 339 nm, typical of the absorption of the A rings (270 nm), B (220), and C (339 nm–observed when the C ring contains unsaturation between carbons C2 and C3) of flavonoids. The presence of flavonoids rutin (t_r_ 16.6 min/λ_max_ = 255 and 354 nm) in Figure 1C, quercetin (t_r_ 23.8 min/λ_max_ = 255 and 371 nm) in Figure 1D, and epicatechin (t_r_ 39.7 min/λ_max_ = 220 and 270 nm) in Figure 1B was determined in the MEMLL (Figure 1A) in the fingerprint of the chromatograms of the standards used. Myricetin (Figure 1E) and kaempferol (Figure 1F) were absent in MEMLL.

#### 3.1.2 Spectroscopy profile by NMR

The spectroscopic profile of the MEMLL was obtained by ^1^H and ^13^C NMR (Supplementary Figures S1, S2), which allowed the evaluation of the chemical composition by identifying the sub-structural fractions (functional groups), as well as evaluating their abundance by normalizing the integrals by spectral regions. Thus, δ_H_ was observed to be 0.5–1.5, which comprises the hydrogen signals of methyl (-CH_3_), methylene (-CH_2_), and methine (-CH) groups, typical of alkyl groups, with approximately 15.79%. The region of the spectrum from δ_H_ 1.5 to 3.0, typical of -CH_3_/-CH_2_/-CH hydrogens, bonded to unsaturated carbon (CH_3_-C=C/CH_2_-C=C/CH-C=C), the ketone carbonyl or aldehyde (CH_3_-COR/CH2-COR/CH-COR), the carboxyl (CH_3_-COOR/CH_2_-COOR/CH-COOR), the nitrogen atoms (CH_3_-N/CH_2_-N/CH-N), or directly linked to the aromatic ring (CH_3_-Ph/CH_2_-Ph/CH-Ph), a percentage area of 10.63%. The region of δ_H_ 3.0 to 4.5, corresponding to the signals of hydrogens bonded to oxidized carbons (CH_2_-OH/CH-OH) or alkoxy, phenoxy, and acylate groups (PhO-CH_3_/PhO-CH_2_/RCOO-CH_3_/RCOO-CH_2_/RCOO-CH), presented the highest percentage area of the studied extract, 62.37%, thus indicating a high sugar composition. Furthermore, in the hydrogen region of olefinic groups, δ_H_ 4.5 to 6.0 (CH_2_ = CH-R/R-CH = CH-R) presented 6.08% of spectrum integration. Another notable region was that of hydrogens linked to aromatic carbons (Ph-H) or olefinic carbons vicinal to the aromatic ring (Ph-CH = CH-R), which showed a higher content of aromatic hydrogens for the 5.13% extract (Supplementary Table S1).

The ^13^C NMR spectrum of the MEMLL was obtained to corroborate the information evidenced in the ^1^H NMR spectrum, having confirmed the presence of signals from aliphatic carbons (-CH_3_, -CH_2_-, -CH -, CH_3_-CO-, and -CH_2_-NH_2_) in the spectral region between δ_C_ 10.0 and 50.0. The region δ_C_ 50.0–90.0, typical of oxidized aliphatic carbons of ether and alcohol (-C-O-C- or -C-OH), showed very intense signals, which highlights the hypothesis of the abundance of sugars, as observed in NMR ^1^H (δ_H_ 3 .0–4.5). For the region of aromatic and olefinic carbons, δ_C_ 100.0–140.0 and between 140.0 and 160.0 indicate oxidized aromatic carbons, highlighting the content of phenolic compounds.

Analysis of two-dimensional NMR spectra (COSY, HSQC, and HMBC) carried out with the MEMLL allowed the evaluation of phytochemical diversity through the observation of correlations ^1^H/^1^H (^2,3^ *J*_H, H_), ^1^H/^13^C (^1^ *J*_C, H_), and ^1^H/^13^C (^2,3^ *J*_C, H_) of the typical signals of bonds and sub-structural fractions present in flavonoids, which allowed the identification of four secondary metabolites: rutin, quercetin, luteolin, and chrysoeriol (Supplementary Figures S3–S5; Supplementary Table S2).

In the homonuclear correlation map COSY ^1^H/^1^H (^2,3^ *J*_H, H_), correlation patterns common to the annotated compounds were observed, such as the related meta-doublets, in δ_H_ 6.20 (*d* 1.9)/6.41 (*d* 1.9); 6.21 (*d* 1.9)/6.45 (*d* 1.9); and 6.19 (*d* 2.7)/6.45 (*d* 2.7), which indicate the presence of 1,2,3,5-tetra-substituted aromatic rings. Another correlation pattern observed indicates the presence of 1,3,4-tri-substituted aromatic rings as follows: δ_H_ 7.53 (*d* 2.4)/6.91 (*d* 7.8)/7.54 (*dd* 2.4; 7.8); 7.55 (*d* 2.3)/6.91 (*d* 8.4)/7.52 (*dd* 2.3; 8.4); and 7.35 (*d* 2.0)/6.82 (*d* 8.3)/7.39 (*dd* 2.0; 8.3). These spectral patterns, in association with the ^13^C NMR spectrum in which carbonyl carbon signals were observed (δ_C_ 177.8; 181.1; 182.9; and 175.7), allow us to relate the sub-structural fractions of flavonoids (rings A and B) (Pinheiro et al., 2022).

In the HSQC spectrum, the correlations between the signals of the aromatic ring sub-structural fraction stand out at δ_H_/δ_C_ 6.20 (*d* 1.9)/99.2; 6.41 (*d* 1.9)/94.1; 7.53 (*d* 2.4)/116.2; 6.91 (*d* 7.8)/116.1; and 7.54 (*dd* 2.4; 7.8)/122.0, two correlated pairs that indicate anomeric H/C at 5.45 (*d* 7.2)/102.3 and 4.4 (*d* 1.6)/100.5; in addition to these, the typical signals of the rhamnose methyl group, at 4.42 (*d* 1.6)/18.4, suggest the presence of a glycosylated flavonoid. After confirming the correlations of the COSY ^1^H/^1^H (^2,3^ *J*_H, H_) and HMBC ^1^H/^13^C (^2,3^ *J*_C, H_) spectra, comparison with literature data (Moura et al., 2011) allowed us to note the structure of the glycosylated flavonoid rutin.

Another set of correlations visualized in the HSQC, COSY, and HMBC spectra, similar to those of the rutin glycoside but with lower intensity, were δ_H_/δ_C_ 6.18 (*d* 2.0)/98.9; 6.40 (*d* 2.0)/94.3; 7.63 (*d* 2.3)/116.3; 6.86 (*d* 8.2)/115.4; and 7.54 (*dd* 8.2; 2.3)/119.5, which allow the presence of the flavonoid quercetin (Cuong et al., 2019).

In the region of hydrogens and aromatic carbons of the HSQC spectrum, correlations are observed at δ_H_/δ_C_ 6.80 (*s*)/102.5; 6.19 (*d* 2.7)/99.2; 6.45 (*d* 2.7)/93.9; 7.35 (*d* 2.0)/113.3; 6.82 (*d* 8.3)/115.9; and 7.39 (*dd* 2.0; 8.3)/119.1. After correlations in the HMBC spectrum and comparison with the literature (Cuong et al., 2019), spectral data were recorded for the compound luteolin. With a spectroscopic profile similar to luteolin, the compound chrysoeriol was noted with spectral data in δ_H_/δ_C_ 7.00 (*s*)/106.6; 6.21 (*d* 1.9)/99.3; 6.45 (*d* 1.9)/94.2; 7.55 (*d* 2.3)/109.7; 6.91 (*d* 8.4)/116.7; and 7.52 (*dd* 2.3; 8.4)/119.4 and the typical sign of a methoxy substituent at 3.59 (*s*)/51.7. In the HMBC spectrum, a correlation between the signal of methoxyl hydrogen at δ_H_ 3.59 and the aromatic carbons at δ_C_ 109.7 (C-2′) was observed, 147.9 (C-4′) and 149.3 (C-3′), thus confirming the position of the methoxyl group on the C-3′ carbon of ring B (Park et al., 2007).

### 3.2 Acute oral toxicity test

Oral administration of the MEMLL at doses of 300 and 2.000 mg/kg did not promote signs of changes in the animals’ body weight gain or food and water consumption (Table 1). There were neither behavioral or physiological changes in the first 4 h of observation nor was any mortality observed in the first 24 h or during the following 14 days. Furthermore, no significant differences in the relative organ masses were observed between the MEMLL groups and the control group (Table 2).

**TABLE 1:** Assessment of body weight gain, food and water consumption, and symptom observation.

Group	Body weight gain (g)	Food consumption (g)	Water consumption (mL)	Symptom observation
Vehicle	2.85 ± 0.11	80.12 ± 0.98	106.7 ± 3.58	Not found
300 mg/kg	2.82 ± 0.08	77.17 ± 2.17	113.3 ± 2.79	Not found
2.000 mg/kg	2.92 ± 0.09	81.18 ± 1.84	110.8 ± 4.17	Not found

Data are expressed as mean ± SEM of six animals per group. No significant differences were found when compared to the control group (one-way ANOVA, followed by Dunnett’s *post hoc* test).

**TABLE 2:** Assessment of the relative weight of organs and death count of animals.

Group	Liver	Kidney	Spleen	Death count
Vehicle	5.20 ± 0.22	1.65 ± 0.11	0.35 ± 0.03	0/6
300 mg/kg	5.58 ± 0.18	1.69 ± 0.08	0.34 ± 0.04	0/6
2.000 mg/kg	5.46 ± 0.18	1.70 ± 0.07	0.38 ± 0.03	0/6

Data are expressed as mean ± SEM of six animals per group. No significant differences were found when compared to the control group (one-way ANOVA, followed by Dunnett’s *post hoc* test).

### 3.3 Evaluation of antinociceptive activity

#### 3.3.1 Acetic acid-induced writhing test

**FIGURE 2:**
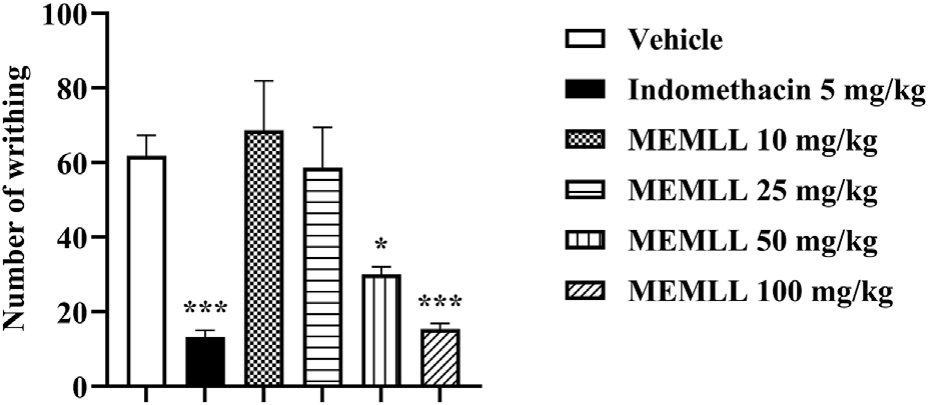
Peripheral analgesic effect of the methanolic extract of MEMLL at 10, 25, 50, and 100 mg/kg in the acetic acid writhing test. Data are expressed as the mean ± SEM of five animals per group. * = *p* < 0.05 and *** = *p* < 0.001 were considered statistically significant when compared to the control group (one-way ANOVA, followed by Dunnett’s *post hoc* test).

The results were obtained with the oral gavage treatment of the MEMLL, as shown in Figure 2. A significant reduction in the percentage of abdominal contortions was observed for the MEMLL at doses of 50 and 100 mg/kg when compared to the control group, 51.46% (*p* < 0.05) and 75.08% (*p* < 0.001), respectively. No reduction in abdominal contortions was observed for MEMLL at doses of 10 and 25 mg/kg. The positive control group with 5 mg/kg indomethacin showed inhibition of 78.64% (*p* < 0.001).

#### 3.3.2 Hot plate test

**FIGURE 3:**
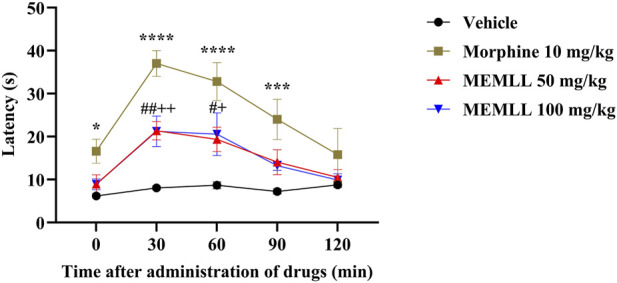
Central analgesic effect of the methanolic extract of MEMLL at 50 and 100 mg/kg in the hot plate test. Data are expressed as the mean ± SEM of five animals per group. * or + or ^#^ = *p* < 0.05, ^++^ or ^##^ = *p* < 0.01, *** = *p* < 0.001, **** = *p* < 0.0001 were considered statistically significant when compared to the control group (two-way ANOVA, followed by Dunnett’s *post hoc* test).

The graph of the hot plate test demonstrates the result obtained when the animals were treated with the MEMLL (Figure 3). The control group maintained low latency at all analysis times. The MEMLL group at a dose of 50 mg/kg was effective when compared to the control group at times of 30 and 60 min, 164.43% (*p* < 0.01) and 122.95% (*p* < 0.05), respectively. Similarly, the MEMLL group at a dose of 100 mg/kg was also effective in increasing latency at times of 30 and 60 min, 162.62% (*p* < 0.01) and 136.68% (*p* < 0.05), respectively. The positive control group with 10 mg/kg morphine showed a longer latency time at 0, 30, 60, and 90 min than the control group, 167.74% (*p* < 0.05), 357.92% (*p* < 0.0001), 277.88% (*p* < 0.0001), and 231.49% (*p* < 0.001), respectively.

#### 3.3.3 Formalin-induced licking response

**FIGURE 4:**
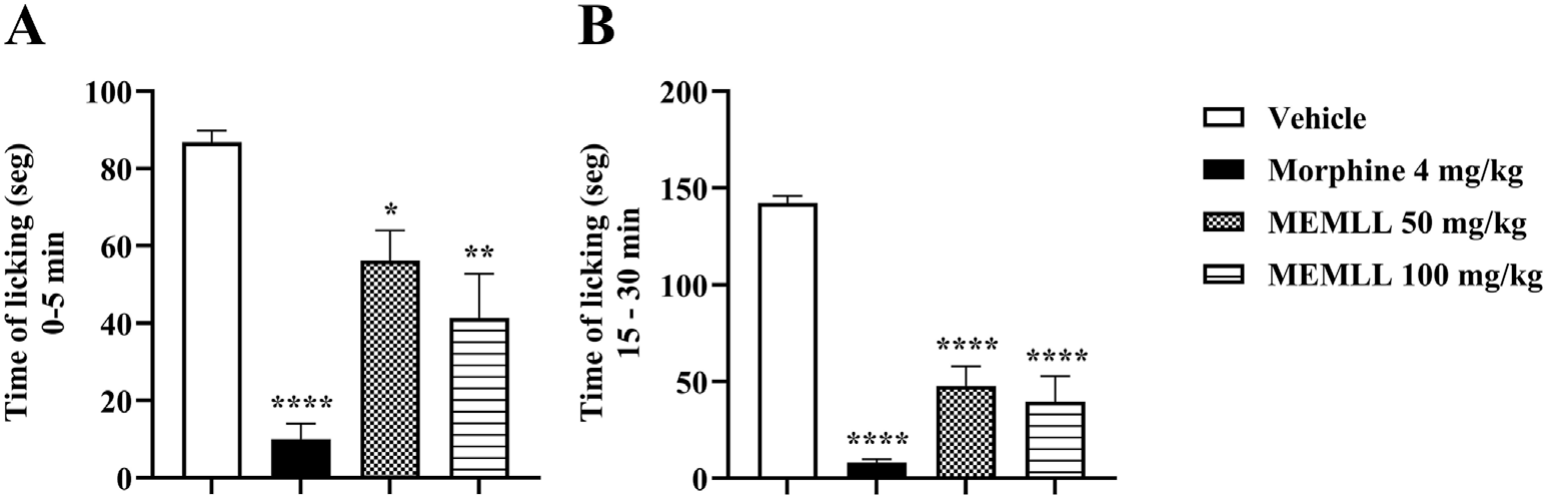
Central and peripheral analgesic effect of the methanolic extract of MEMLL at doses of 50 and 100 mg/kg in the formalin test. **(A)** First phase assesses neurogenic pain. **(B)** Second phase assesses inflammatory pain. Data are expressed as the mean ± SEM of five animals per group. * = *p* < 0.05, ** = *p* < 0.01, and **** = *p* < 0.0001 were considered statistically significant when compared to the control group (one-way ANOVA, followed by Dunnett’s *post hoc* test).

The formalin test graph shows the results obtained when the animals were treated with MEMLL in Figure 4. The groups treated with the MEMLL showed promising results, suppressing paw licking activity in both the first and second phases. The antinociceptive effect in the neurogenic phase occurs with inhibition in the paw licking time of mice treated with the MEMLL at doses of 50 and 100 mg/kg compared to the control group, 35.25% (*p* < 0.05) and 52.30% (*p* < 0.01), respectively. In the inflammatory phase, inhibition was also observed in the MEMLL at doses of 50 and 100 mg/kg, 66.39% (*p* < 0.0001) and 72.15% (*p* < 0.0001), respectively. The positive control with 4 mg/kg morphine was effective in both the neurogenic phase (88.48%, *p* < 0.0001) and the inflammatory phase (94.37%, *p* < 0.0001).

## 4 Discussion

Natural products are widely accepted and recognized as effective therapeutics for various diseases and drug development (Umeh et al., 2024). Historically, natural products have provided a wide range of chemical molecules and remain one of the main sources of new and effective modern drugs (Nahar and Sarker, 2020). In this work, we used the methanolic extract after performing a bioassay-guided fractionation analysis (data not shown). So, the current investigation aimed to evaluate the ethnopharmacological use of *M. linifera* regarding its antinociceptive potential. For this purpose, chemical characterization, acute oral toxicity test, and nociceptive tests on MEMLL were carried out.

The HPLC fingerprint analysis identified three phenolic compounds classified as flavonoids: rutin, quercetin, and epicatechin. Similarly, to corroborate the study, the analysis of NMR spectra allowed the identification of rutin and quercetin again, as well as luteolin and chrysoeriol. Plant secondary metabolism produces flavonoids with several pharmacological effects (Liu et al., 2024). In short, rutin is known for biological activities such as its anticarcinogenic, antioxidant, anti-inflammatory, antinociceptive, cardioprotective, and neuroprotective effects (Ganeshpurkar and Saluja, 2017). Quercetin has antioxidant, anti-inflammatory, and antinociceptive effects (Azevedo et al., 2013). Epicatechin is reported to have antidiabetic, antioxidant, anti-inflammatory, antinociceptive, and neuroprotective activities (Qu et al., 2021). Luteolin has anticarcinogenic, antioxidant, anti-inflammatory, and antinociceptive effects (Hara et al., 2014). Chrysoeriol has antiarthritic, anticancer, antidiabetic, antioxidant, anti-inflammatory, and antinociceptive properties (Law et al., 2024). The chemical compounds found may contribute to the antinociceptive activities of the MEMLL.

In the present study, we report acute oral toxicity in the species *M. linifera* for the first time. The use of medicinal plants has become an increasingly common practice due to the low cost and high consumption of natural products by society. However, there is still a belief that medicinal plants do not have toxic effects, and therefore, it is necessary to conduct research to obtain data on their safety (Araldi et al., 2021). The acute oral toxicity test is an experiment that precedes the execution of pharmacological tests. It is important to obtain preliminary data on the toxic effects of a substance and, therefore, avoid harm when administered.

The results demonstrate that no effects are considered toxic; the MEMLL lethal dose (LD_50_) is greater than 2.000 mg/kg. According to OECD guideline 423, drugs that have LD_50_ between 2.000 and 5.000 mg/kg are classified as category 5, presenting low acute oral toxicity (OECD, 2002). Pereira et al. (2022) performed cytotoxicity tests on tumor cell lines, with the MEMLL collected in different locations on the Píaui Coast, and it was observed that the methanolic extract of the leaves showed high antiproliferative effects on the B16-F10 cell line (murine metastatic melanoma). Therefore, further studies are needed to understand the anticarcinogenic potential of *M. linifera* leaves as our study sought to evaluate acute oral toxicity in healthy animals.

Furthermore, we evaluated the peripheral and central antinociceptive effects of MEMLL in the acetic acid, hot plate, and formalin tests. The acetic acid writhing test is used to assess the inflammatory-based analgesic activity of a potential drug (de Jesus et al., 2023). In this nociception model, the intensity of the painful stimulus is proportional to the number of abdominal writhings (da Fonseca et al., 2021).The physical writhing response involves the conversion of arachidonic acid to eicosanoids by lipoxygenases (LOX) and cyclooxygenases (COX), which cause hyperalgesia by sensitizing peripheral pain neurons (Alqudah et al., 2023; Oqal et al., 2023). Eicosanoids are responsible for cellular signaling and inflammatory mediators by releasing prostaglandins, mainly prostaglandin E2 (PGE2), which are related to pathological circumstances (Silva et al., 2023).

The antinociceptive activities of the acetic acid test are closely linked to the MEMLL compounds since all the flavonoids have anti-inflammatory activities and may have inhibited nociception by suppressing peripheral LOX and COX levels, reducing the production of inflammatory mediators such as PGE2. This is the first report of a dose-related inhibition curve of an extract from *M. linifera*. In addition, the result of the effect with MEMLL corroborates a previous initial study found in the literature, in which a dose of 400 mg/kg of the ethanolic extract of *M. linifera* leaves was used and also obtained a reduction in peripheral pain (Silva et al., 2013).

The hot plate test is a widely used model to evaluate antinociceptive effects on the central nervous system, such as opioids, through thermal stimulation (Asiri et al., 2024). Animals are placed on a hot plate at a fixed temperature, where the response time of licking one of the paws or jumping is observed, which requires coordination of the central nervous system (Xie et al., 2023). In this test, opioid drugs increase the nociception threshold of rodents in relation to heat via spinal and supraspinal receptors (Manouze et al., 2017). Therefore, the MEMLL showed central antinociceptive effects linked to the flavonoids’ properties and their probable action on opioid receptors. This is the first report of the species *M. linifera* with the hot plate test.

The formalin test was performed to support the central and peripheral analgesic effects of the MEMLL. This model is widely used in rodents, causing painful stimuli in the intraplantar region of the animal’s hind paw. The licking is perceived after injection of the chemical substance that corresponds to the painful response (Hirota et al., 2023). The first phase, which evaluates neurogenic pain, occurs through direct formalin stimulation in primary afferent nociceptors, releasing bradykinin and substance P (Santos et al., 2024). The second phase of inflammatory pain occurs through the release of inflammatory mediators at the site of the injury, such as prostaglandins, serotonin, and bradykinin (Todorov et al., 2024). The central and peripheral analgesic activity of the MEMLL was observed in the formalin test, corroborating the antinociceptive effects observed in the hot plate test and acetic acid test, respectively. This is the first report on the species *M. linifera* with the formalin nociception test.

Finally, the data support the use of *M. linifera* in folk medicine for the treatment of pain. The current study is a significant step toward the sustainable development of family farming as it highlights the potential of the species as a source of income due to its traditional use. Thus, we propose that a more detailed investigation of the species *M. linifera* will provide information to determine the specific pathways and its use as a new therapeutic agent for pain management in the future.

## 5 Conclusion

The flavonoids identified in the MEMLL showed analgesic activities in nociceptive tests, validating the Brazilian ethnopharmacological use of this plant for pain treatment. The MEMLL did not demonstrate toxic effects or mortality, highlighting its safety profile. In this important work, we present the first report on these compounds identified in the chemical characterization of the leaves of the species *M. linifera*. This is a significant contribution to the ethnopharmacology field. Furthermore, we are the first to evaluate the acute oral toxicity, dose-related inhibition in the acetic acid test, and antinociceptive effects in the hot plate and formalin tests in the leaves of the species *M. linifera*, which are of great interest to the Amazon community.

## Data Availability

The raw data supporting the conclusion of this article will be made available by the authors, without undue reservation.
