# Supplementary material for: Chemical characterization, assessment of acute oral toxicity, and antinociceptive potential of the methanolic extract of Montrichardia linifera (Arruda) Schott leaves from Brazil
Source: Front Pharmacol. 2024 Nov 20;15:1475157. doi: 10.3389/fphar.2024.1475157 (PMC11615642; doi:10.3389/fphar.2024.1475157)
Supplement: Supplementary file 1 [file DataSheet1.DOCX]

Supplementary Material

# Supplementary Figures and Tables

## Supplementary Figures


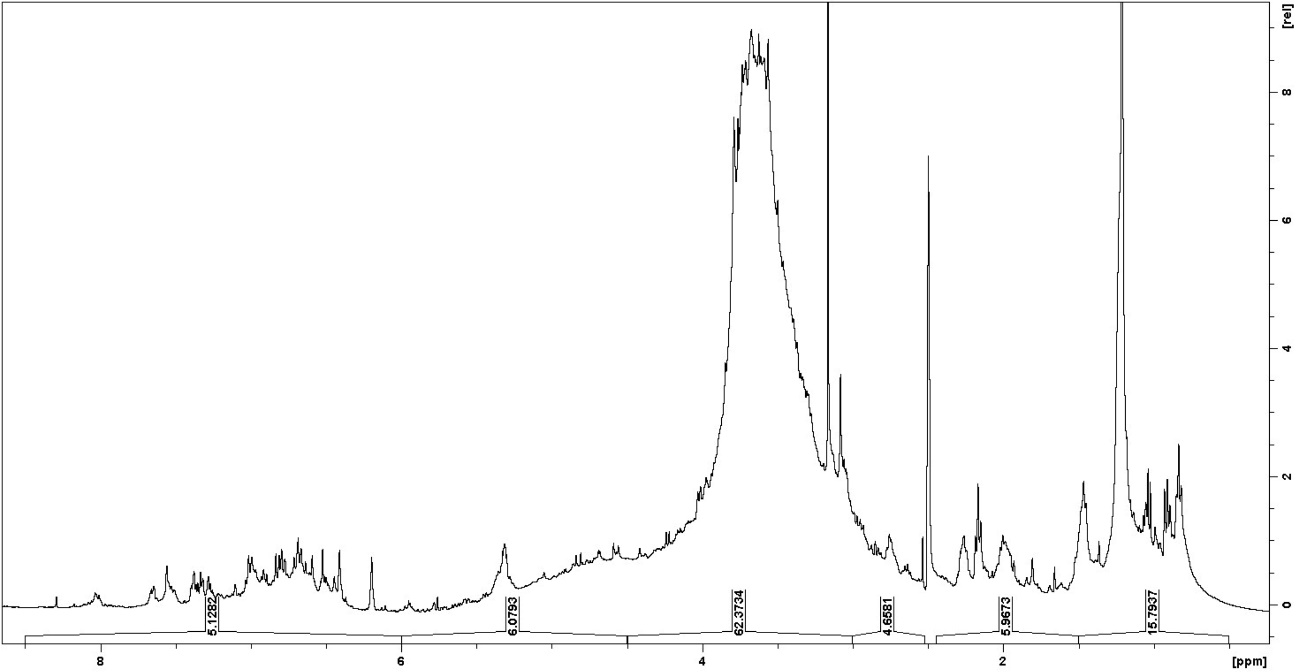


Supplementary Figure 1. ^1^H NMR spectrum of the MEMLL.


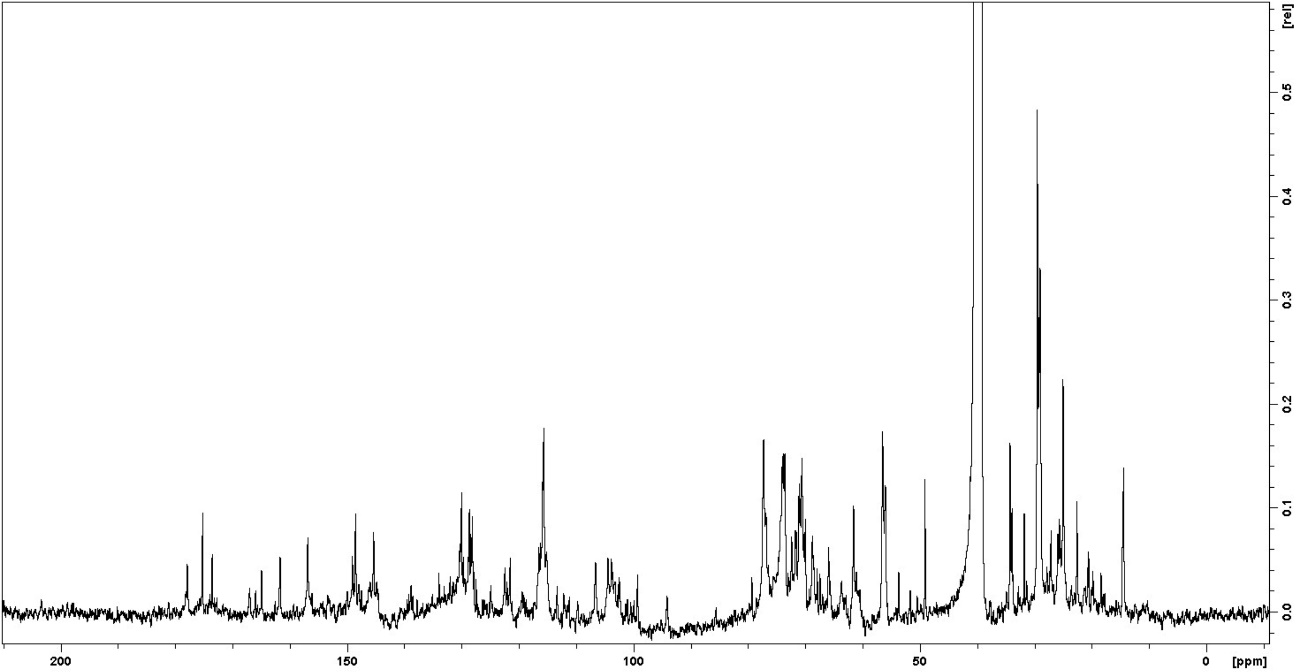


Supplementary Figure 2. ^13^C NMR spectrum of the MEMLL.


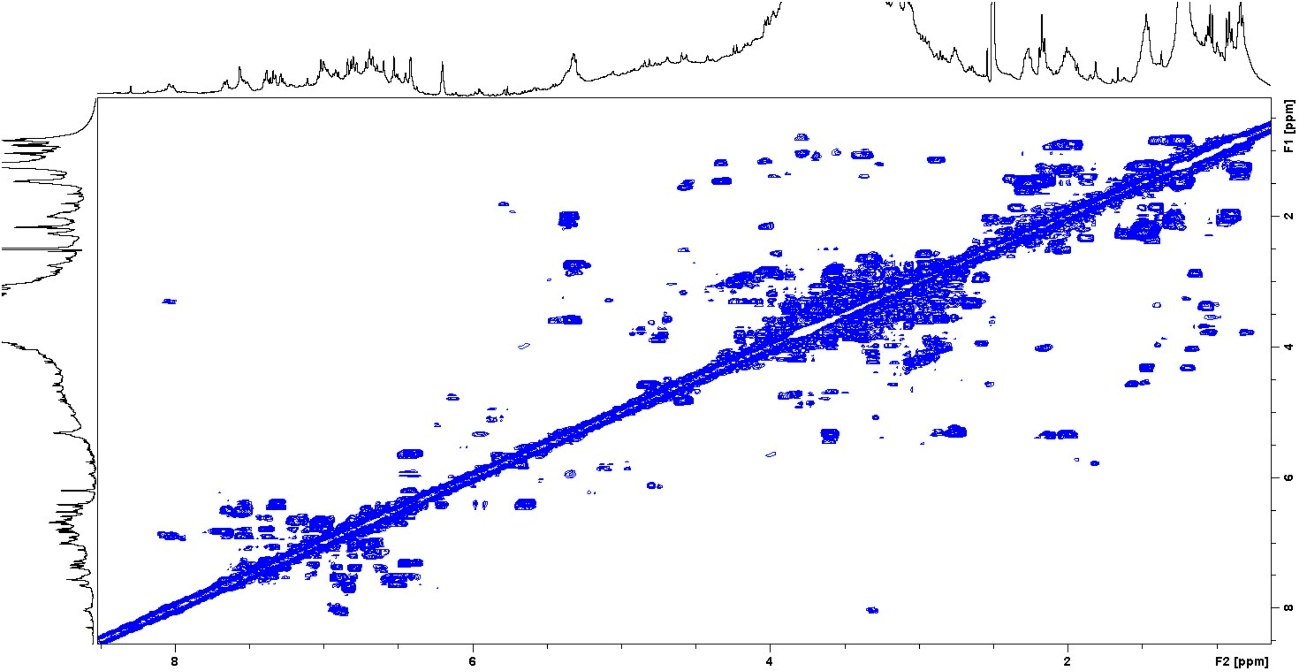


Supplementary Figure 3. HOMO-COSY NMR spectrum correlation map from the MEMLL.


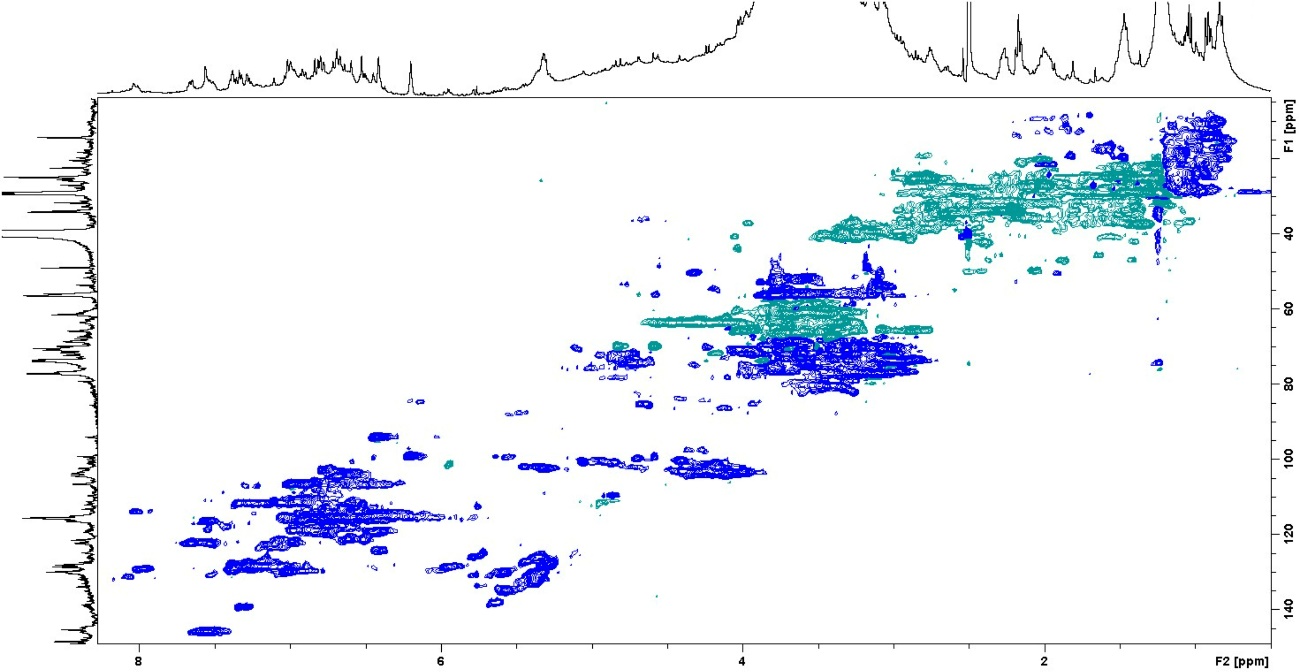


Supplementary Figure 4. HSQC NMR spectrum correlation map from the MEMLL.


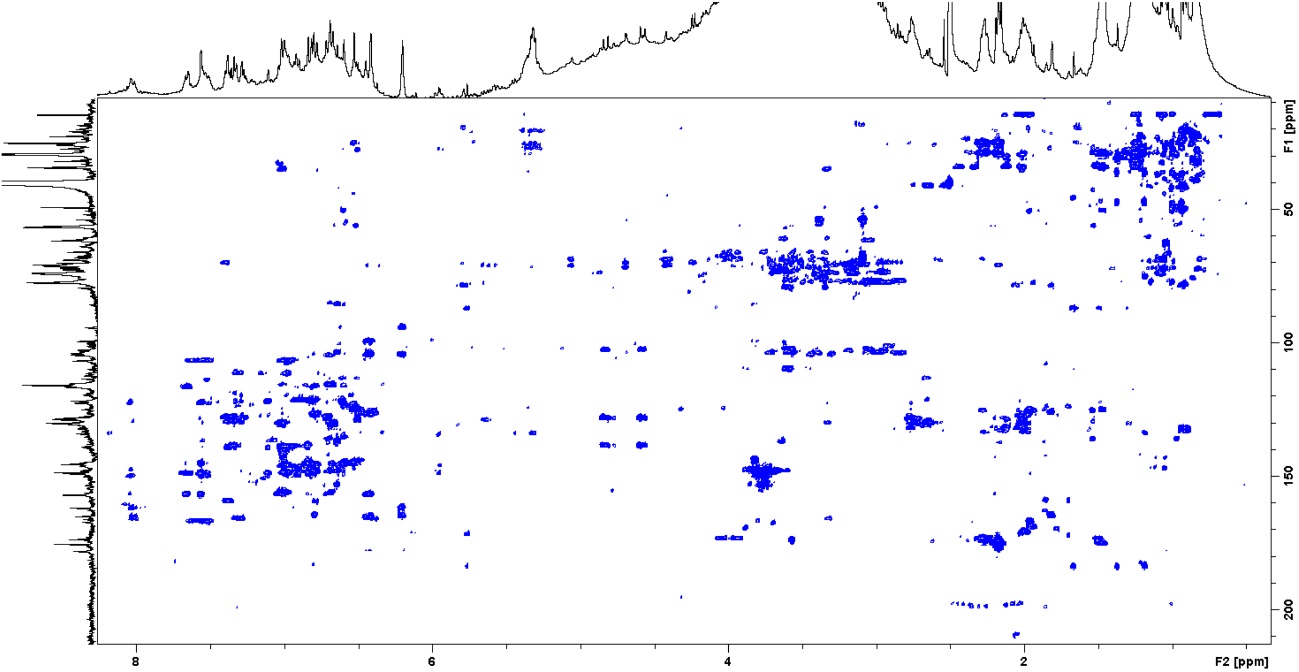


Supplementary Figure 5. HMBC NMR spectra correlation map from the MEMLL.

## Supplementary Tables

Supplementary Table 1. Characterization of the MEMLL by ^1^H NMR.

| Chemical shift (ppm)* | Assignments | Area (%) |
| --- | --- | --- |
| 0.5 – 1.5 | C-C**H**_n_ | 15.79 |
| 1.5 – 3.0 | C**H**_n_-C=C; C**H**_n_-COR; C**H**_n_-COOR; C**H**_n_-N; C**H**_n_-Ph | 10.63 |
| 3.0 – 4.5 | C**H**_n_-OH; PhO-C**H**_n_; RCOO-C**H**_n_; C**H**_2_-NHCOR | 62.37 |
| 4.5 – 6.0 | C**H**_n_=CH | 6.08 |
| 6.0 – 9.0 | Ph-**H**; Ph-C**H**=CH-R | 5.13 |

Legend: n = number of hydrogens, in which 1 ≤ n ≤ 3. * Pinheiro et al. (2022)

Supplementary Table 2. Assignment of metabolites in extracts of M. linifera by NMR.

| ID | Compounds | Position | δ ^1^H (*J*/Hz) | δ ^13^C | Correlations | |
| --- | --- | --- | --- | --- | --- | --- |
|  |  |  |  |  | COSY | HMBC |
| 1 | Rutin |  |  |  |  |  |
|  |  | 2 | - | 156.8 | - | - |
|  |  | 3 | - | 133.9 | - | - |
|  |  | 4 | - | 177.8 | - | - |
|  |  | 5 | - | 161.6 | - | - |
|  |  | 6 | 6.20 (*d* 1.9) | 99.2 | 6.41 | 161.6; 164.9 |
|  |  | 7 | - | 164.9 | - | - |
|  |  | 8 | 6.41 (*d* 1.9) | 94.1 | 6.20 | 156.6; 164.9 |
|  |  | 9 | - | 156.6 | - | - |
|  |  | 10 | - | 103.8 | - | - |
|  |  | 1’ | - | 121.5 | - | - |
|  |  | 2’ | 7.53 (*d* 2.4) | 116.2 | 7.54 | 121.5; 145.3; 149.0 |
|  |  | 3’ | - | 145.3 | - | - |
|  |  | 4’ | - | 149.0 | - | - |
|  |  | 5’ | 6.91 (*d* 7.8) | 116.1 | 7.54 | 121.5; 145.3; 149.0 |
|  |  | 6’ | 7.54 (*dd* 2.4; 7.8) | 122.0 | 6.91; 7.53 | 116.1; 121.5; 149.0 |
|  |  | 1” | 5.45 (*d* 7.2) | 102.3 | - | 133.9 |
|  |  | 1’” | 4.42 (*d* 1.6) | 100.5 | - | - |
|  |  | 6’” | 4.42 (*d* 1.6) | 18.4 | - | - |
| 2 | Chrysoeriol |  |  |  |  |  |
|  |  | 2 | - | 162.5 | - | - |
|  |  | 3 | 7.00 (*s*) | 106.6 | - | 122.6; 162.5; 181.1 |
|  |  | 4 | - | 181.1 | - | - |
|  |  | 5 | - | 162.6 | - | - |
|  |  | 6 | 6.21 (*d* 1.9) | 99.3 | 6.45 | 162.6; 165.6 |
|  |  | 7 | - | 165.6 | - | - |
|  |  | 8 | 6.45 (*d* 1.9) | 94.2 | 6.21 | 103.6; 157.2; 165.6 |
|  |  | 9 | - | 157.2 | - | - |
|  |  | 10 | - | 103.6 | - | - |
|  |  | 1’ | - | 122.6 | - | - |
|  |  | 2’ | 7.55 (*d* 2.3) | 109.7 | 7.52 | 119.4; 122.6; |
|  |  | 3’ | - | 149.3 | - | - |
|  |  | 4’ | - | 147.9 | - | - |
|  |  | 5’ | 6.91 (*d* 8.4) | 116.7 | 7.52 | 119.4; 147.9 |
|  |  | 6’ | 7.52 (*dd* 2.3; 8.4) | 119.4 | 6.91 | 116.7; 147.9 |
|  |  | OMe | 3.59 (*s*) | 51.7 | - | 109.7; 147.9; 149.3 |
| 3 | Luteolin |  |  |  |  |  |
|  |  | 2 | - | 164.6 | - | - |
|  |  | 3 | 6.80 (*s*) | 102.5 | - | 122.1; 164.6; 182.9 |
|  |  | 4 | - | 182.9 | - | - |
|  |  | 5 | - | 161.8 | - | - |
|  |  | 6 | 6.19 (*d* 2.7) | 99.2 | 6.45 | 93.9; 161.8; 164.9 |
|  |  | 7 | - | 164.9 | - | - |
|  |  | 8 | 6.45 (*d* 2.7) | 93.9 | 6.19 | 99.2; 103.5; 156.7 |
|  |  | 9 | - | 156.7 | - | - |
|  |  | 10 | - | 103.5 | - | - |
|  |  | 1’ | - | 122.1 | - | - |
|  |  | 2’ | 7.35 (*d* 2.0) | 113.3 | 7.39 | 122.1; 145.4; 149.9 |
|  |  | 3’ | - | 145.4 | - | - |
|  |  | 4’ | - | 149.9 | - | - |
|  |  | 5’ | 6.82 (*d* 8.3) | 115.9 | 7.39 | 119.1; 149.9 |
|  |  | 6’ | 7.39 (*dd* 2.0; 8.3) | 119.1 | 6.82; 7.35 | 122.1; 115.9 |
| 4 | Quercetin |  |  |  |  |  |
|  |  | 2 | - | 146.2 | - | - |
|  |  | 3 | - | 135.8 | - | - |
|  |  | 4 | - | 175.7 | - | - |
|  |  | 5 | - | 160.4 | - | - |
|  |  | 6 | 6.18 (*d* 2.0) | 98.9 | 6.40 | 160.4; 164.3 |
|  |  | 7 | - | 164.3 | - | - |
|  |  | 8 | 6.40 (*d* 2.0) | 94.3 | 6.18 | 103.2; 156.1; 164.3 |
|  |  | 9 | - | 156.1 | - | - |
|  |  | 10 | - | 103.2 | - | - |
|  |  | 1’ | - | 121.6 | - | - |
|  |  | 2’ | 7.63 (*d* 2.3) | 116.3 | 7.54 | 115.4; 145.0; 148.0 |
|  |  | 3’ | - | 145.0 | - | - |
|  |  | 4’ | - | 148.0 | - | - |
|  |  | 5’ | 6.86 (*d* 8.2) | 115.4 | 7.54 | 119.5; 145.0; 148.0 |
|  |  | 6’ | 7.54 (*dd* 8.2; 2.3) | 119.5 | 6.86; 7.63 | 146.2; 160.4 |
